# A bacterial toxin–antitoxin system as a native defence element against RNA phages

**DOI:** 10.1098/rsbl.2025.0080

**Published:** 2025-06-11

**Authors:** Nela Nikolic, Maroš Pleška, Tobias Bergmiller, Călin C. Guet

**Affiliations:** ^1^ Institute of Science and Technology Austria, Klosterneuburg, Austria; ^2^ Faculty of Health and Life Sciences, Living Systems Institute, University of Exeter, Exeter, UK; ^3^ Faculty of Environment, Science and Economy, Department of Physics and Astronomy, University of Exeter, Exeter, UK; ^4^ Faculty of Environmental and Life Sciences, School of Biological Sciences, University of Southampton, Southampton, UK; ^5^ The Rockefeller University, New York, NY, USA; ^6^ New York Genome Center, New York, NY, USA; ^7^ Faculty of Health and Life Sciences, Department of Biosciences, University of Exeter, Exeter, UK

**Keywords:** *Escherichia coli*, RNA phage, toxin–antitoxin system, antiphage defence, bacteria–phage interactions, phenotypic heterogeneity

## Abstract

Bacteria have evolved a wide range of defence strategies to protect themselves against bacterial viruses (phages). Most known bacterial antiphage defence systems target phages with DNA genomes, which raises the question of how bacteria defend against phages with RNA genomes. Bacterial toxin–antitoxin systems that cleave intracellular RNA could potentially protect bacteria against RNA phages, but this has not been explored experimentally. In this study, we investigated the role of a model toxin–antitoxin system, MazEF, in protecting *Escherichia coli* against two RNA phage species. When challenged with these phages, the native presence of *mazEF* moderately reduced population susceptibility and increased the survival of individual *E. coli* cells. Genomic analysis further revealed an underrepresentation of the MazF cleavage site in genomes of RNA phages infecting *E. coli*, indicating selection against cleavage. These results show that, in addition to other physiological roles, RNA-degrading toxin–antitoxin systems may also help defend against RNA phages.

## 1. Introduction

Bacterial and archaeal viruses (phages) are key drivers of prokaryotic evolution. The known phage defence mechanisms, including cell surface modifications, restriction–modification, CRISPR-Cas and abortive infection systems, almost exclusively target phages with DNA genomes [1–5]. This raises the question of how and if at all, bacteria can defend against RNA phages. Although no direct defence mechanisms against RNA phages have so far been described, several toxin–antitoxin (TA) systems have been hypothesized to cleave intracellular single-stranded RNA (ssRNA) under specific conditions [6,7], suggesting they might potentially defend against RNA phages. While this has not been shown experimentally and the exact mechanism by which toxins are activated remains debated [8], TA systems are primarily known for other cellular functions, such as protecting bacteria from invading plasmids, modulating gene expression and increasing stress tolerance [6,9–11]. TA systems have also been implicated in defending against DNA phages either through interference with phage replication or through promoting abortive infection [12,13]. Abortive infection systems trigger bacterial cell death or metabolic arrest upon phage infection, causing lysis or dormancy before the phage completes its replication cycle. However, to what extent and by what mechanism, TA systems protect bacteria against RNA phages remains an open question.

Here we investigate the role of the TA module *mazEF* in defending *E. coli* against MS2 (*Emesvirus zinderi*) and Qβ (*Qubevirus durum*)—two of the best-studied RNA phages. MazEF is one of the most abundant TA systems in *E. coli*, present in over 80% of sequenced strains [14,15]. Toxin MazF recognizes the ACA trinucleotide in ssRNA [16–18]. Antitoxin MazE binds to MazF to neutralize its RNA-degrading activity or acts as a repressor of *mazEF* expression. MS2 and Qβ are positive-sense ssRNA ((+)ssRNA) phages, meaning their genomes serve directly as mRNA for protein translation [19]. Experimentally determined secondary structures of MS2 and Qβ genomes indicate that ACA trinucleotides can be found in unpaired segments of the loop regions, i.e. single-stranded regions accessible to MazF cleavage [20,21]. The MS2 RNA genome has been used to assess the *in vitro* RNA cleavage activity of MazF in *E. coli* [17] and in other species [22–25]. To what extent such cleavage occurs *in vivo* is unknown.

## 2. Methods

### (a) Growth assays

Cultures of *E. coli* strain K-12 MG1655 and its derivatives harbouring F plasmid were grown at 37°C (electronic supplementary material, table S1 and supplementary methods). Overnight cultures of the wild-type (wt) NN239 F+ and Δ*mazEF* NN241 F+ strains were diluted 1 : 1000 into 4 ml of LB medium. After 3 h, cultures were supplemented with 0.01% glucose and 2 mM CaCl_2_ and 195 μl of the cultures were put into a 96-well plate. Cultures were then infected with 5 μl of the RNA phage lysate. SM buffer was added to uninfected cultures. Absorbance at 600 nm A_600_ was recorded every 5 min for 10 h using a CLARIOstar plate-reader (BMG Labtech).

### (b) Competition assays

Exponentially growing cultures of the competing strains were mixed in 1 : 1 ratio and split into two parts: the first part remained uninfected, while the second part was infected with RNA phage. Samples were incubated at 37°C without shaking for 27 and 15 min for MS2 and Qβ, respectively, then incubated at 37°C with shaking for a total time of 90 and 105 min for MS2 and Qβ, respectively. Following incubation, all samples were washed with SM buffer to remove non-adsorbed phage and re-suspended in fresh medium. Serial dilutions were plated on tetrazolium arabinose agar plates (1% tryptone, 0.1% yeast extract, 1% arabinose, 0.5% NaCl, 0.005% triphenyltetrazolium chloride, 1.6% agar), then incubated for 24 h. The Wrightian fitness of the wt strain at time *t* was determined relative to the fitness of the Δ*mazEF* strain as (CFUwt*_t_*/CFUwt_0_)/(CFUΔ*_t_*/CFUΔ_0_). The *araA* mutation was used as a neutral marker for competition experiments [26].

### (c) Single-cell assays

For microscopy experiments, we used the wt NN242-cat F+ and Δ*mazEF* NN243-cat F+ *E. coli* strains that carry chromosomally encoded *mCherry* fluorescent reporter gene. Microfluidic devices [27,28] were operated using NE-700 syringe pumps with a constant flow rate of 2 ml h^−1^. A temperature-controlled Olympus IX83 microscope was equipped with a Lumencore SpectraX light source and a custom-made autofocus system [29]. Images were acquired every 5 min using a 100 × 1.4 NA oil immersion objective and a cooled Photometrics Prime95B. To image mCherry, we used the green LED (549 ± 15 nm) with an intensity of 320 mW and an exposure time of 200 ms. To image fluorescein, we used the cyan LED (475 ± 28 nm) at 180 mW and 25 ms exposure time. Emission filters were from Semrock (LP 495, BP 520/35 for GFP and LP 596, BP 641/75 for mCherry). As F-pili promote biofilm formation in *E. coli* F+ strains [30], overnight cultures were mixed with 0.1% Tween to enable efficient loading of bacteria into the microfluidic device and to prevent bacteria from clumping within the device. Loaded bacteria were grown for at least 3 h at 37°C in phage-free medium to reach a steady-state growth, before switching to medium supplemented with 0.01% glucose, 2 mM CaCl_2_ and 0.001% fluorescein, and containing phage lysate in the final concentration of 10^9^ MS2 or Qβ phage particles ml^−1^. Fluorescein was used to determine the exact timing of the media switching and exposure of bacterial cells to phage. Cells at the blunt end of each growth channel were analysed during 650 min of MS2 infection or 985 min of Qβ infection, and phenotypes were confirmed by manual analysis (electronic supplementary material, supplementary methods).

### (d) Genome analysis

First, we collected 12 full-length reference genomes of viruses with (+)ssRNA genomes that infect Bacteria (RNA phages) from the National Center for Biotechnology Information’s (NCBI) Viral Genomes Database [31], https://www.ncbi.nlm.nih.gov/genome/viruses/ (March 2019). We also collected 2216 genomes of phages with genomes other than (+)ssRNA, and 1835 genomes of (+)ssRNA viruses that do not infect Bacteria. Second, from the NCBI Nucleotide Database https://www.ncbi.nlm.nih.gov/nucleotide/, we collected 28 RNA phage genomes described in [32], 20 RNA phage genomes described in [33] and an additional 33 complete and partial RNA phage genomes with a length of more than 1000 nucleotides and excluding genomes obtained from experimentally evolved strains. This resulted in a total of 81 RNA phage genomes (electronic supplementary material, table S2). Of these 81 phages, 57 were (+)ssRNA phages that infect *Escherichia*, excluding ‘unclassified’ members.

ACA sites (ACA trinucleotides) were counted in every genome, and the frequency of ACA sites was calculated as [*n*_ACAgenome_/(length_genome_ – 2)]. Relative ACA frequency was calculated as the frequency of actual ACA sites divided by the expected frequency. Expected ACA frequency was calculated as [fraction(A)_genome_ * fraction(C)_genome_ * fraction(A)_genome_]. Number of expected ACA sites was calculated as [fraction(A)_genome_ * fraction(C)_genome_ * fraction(A)_genome_ * length_genome_]. Analysis of other toxin recognition trinucleotides—GCU (MqsR recognition sequence), ACG and ACU (ChpB recognition sequences)—was done analogously. Each ssRNA phage genome was shuffled such that the nucleotide content remained the same as in the original genome (fractions of A, C, G and U were the same in the original and shuffled genomes), and shuffling of nucleotides was repeated 10 000 times.

## 3. Results

### (a) *mazEF* increases population fitness during exposure to RNA phages

**Figure 1.**
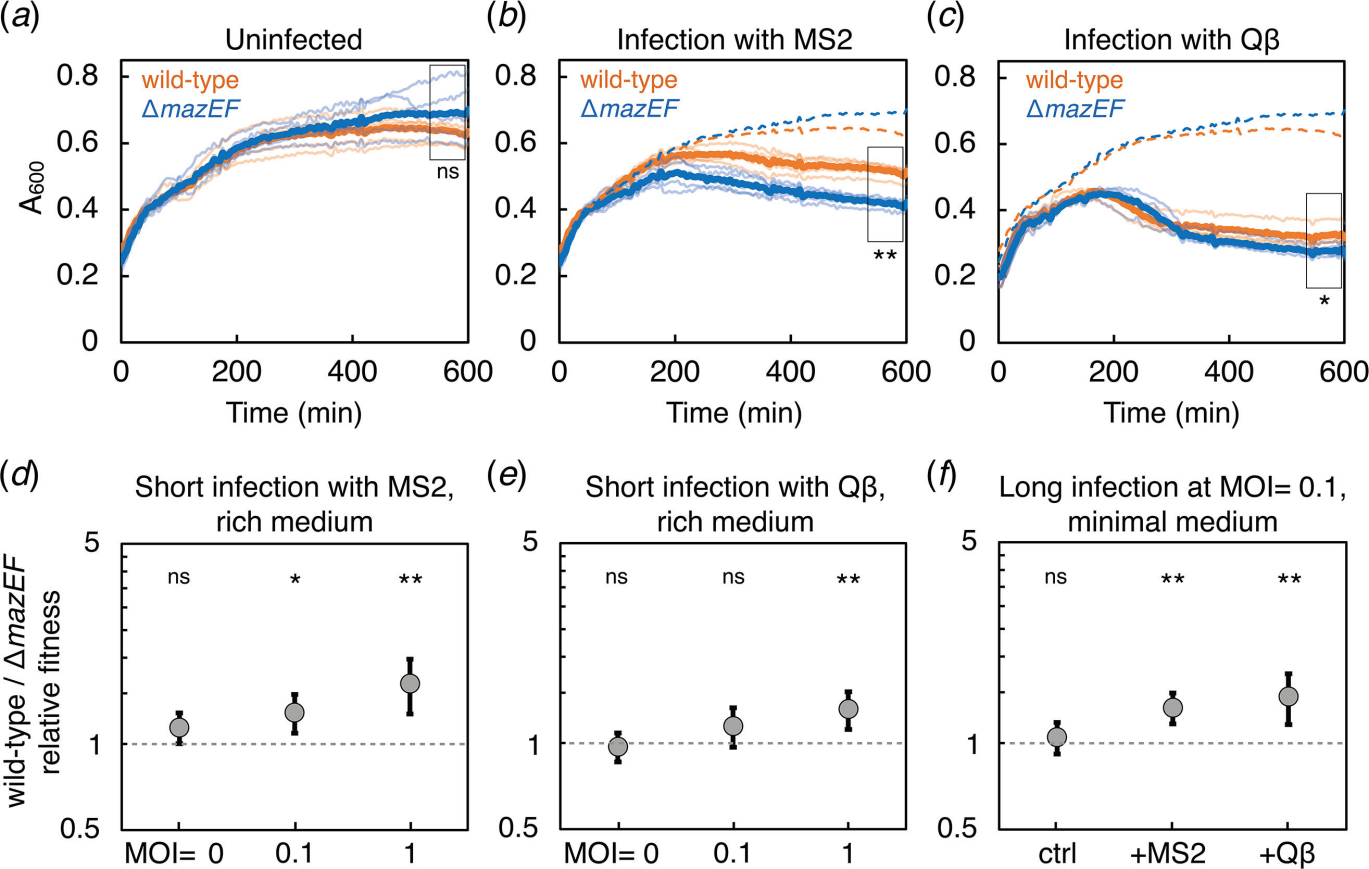
Population growth in response to RNA phage infection. (*a*) Growth curves of uninfected wt (orange line) and Δ*mazEF* (blue line) cultures. Population growth after (*b*) MS2 or (*c*) Qβ infection at MOI = 1. Pale lines represent individual replicates (five replicates per treatment). Bold lines show averages across replicates. Dashed lines represent the mean values of uninfected cultures. *p*-values were determined using *t*-tests performed on the highlighted data, **p < 0.01, *p < 0.05, ns, non-significant. (*d*) Short-term competition experiments between wt and Δ*mazEF* strains during MS2 (25 control replicates, 13 replicates at MOI = 0.1, 12 replicates at MOI = 1) or (*e*) Qβ infection (12 control replicates, six replicates at MOI = 0.1, six replicates at MOI = 1). (*f*) Long-term competition experiments (*t* = 20 h; 40 control replicates, 25 replicates infected with MS2, 18 replicates infected with Qβ). *p*-values were calculated by fitting a linear regression model with the null hypothesis that the average relative fitness is equal to 1 (dashed line). Strain labelling does not affect relative fitness, *p* = 0.23. Error bars show 95% confidence intervals.

We first measured the effect of MS2 and Qβ on wt *E. coli*, which natively encodes *mazEF*, and compared it to isogenic strains with a deleted *mazEF* locus (Δ*mazEF*) during the exponential growth phase. Since phages MS2 and Qβ initiate infection by binding along the side of F-pili [19], all *E. coli* strains in this study carry the F plasmid encoding the pili. At the initial multiplicity of infection (MOI, the ratio of phage particles to bacterial cells in a culture) of 1, the biomass of the wt *E. coli* batch cultures 10 h post-infection was on average 22% (18% for Qβ) larger than that of Δ*mazEF* cultures (figure 1*a*–*c*; electronic supplementary material, figure S1). In plating experiments, native *mazEF* presence significantly increased the number of colony forming units (CFU) following phage infection at MOI = 0.01 (1.7- and 2.5-fold increase for MS2 and Qβ, respectively), with the effect being more pronounced at higher MOIs (electronic supplementary material, figure S2). In a synthetic overexpression system, chemical induction of *mazF* expression increased survival of cells exposed to phage from 1% to 68% for MS2 and from 13% to 56% for Qβ (electronic supplementary material, figure S3). However, this larger effect could be predominantly non-specific and caused by the MazF-induced growth arrest rather than direct interference [28]. The *mazEF* deletion did not affect RNA phage adsorption, but mildly increased the production of progeny phage particles (electronic supplementary material, figures S4 and S5). This indicates that rather than lowering the likelihood of phage adsorption (which could occur, for example, due to lower expression of F-pili), MazEF interferes with RNA phage replication within already infected cells.

A significant effect of *mazEF* was also detected in direct competition assays, where mixed wt and Δ*mazEF* cultures were exposed to phage for the duration corresponding to a single phage replication cycle (90 and 105 min for MS2 and Qβ, respectively) [34–37]. The relative fitness of the wt strain was 1.29 and 1.15 at MOI = 0.1 for MS2 and Qβ, with increased effects observed at an increased MOI (figure 1*d*,*e*) or with a prolonged incubation time (figure 1*f*; electronic supplementary material, supplementary methods and supplementary datasets). Importantly, deletion of *mazEF* did not significantly affect fitness in the absence of the phage. Overall, the direct competition experiments show that cells with native *mazEF* have an increased probability of surviving an RNA phage infection compared to Δ*mazEF* cells.

### (b) *mazEF* delays time to lysis and increases size of individual bacterial cells challenged with RNA phage

**Figure 2.**
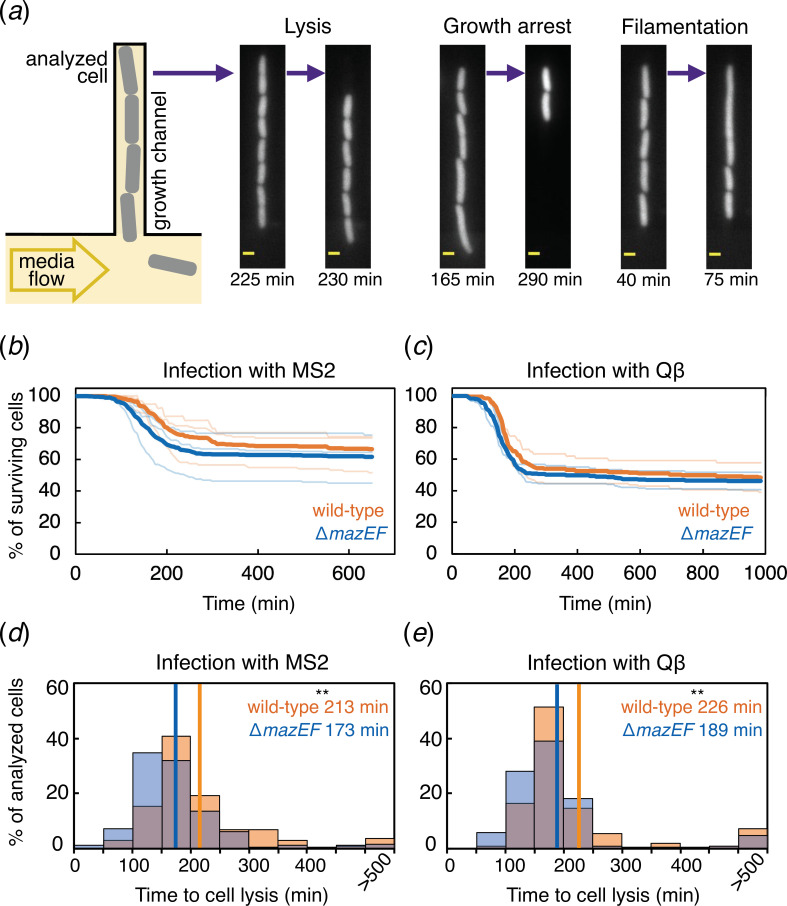
Single-cell analysis of RNA phage infection. (*a*) Schematic of microfluidic device and representative fluorescence microscopy images. Lysis of cells manifested as loss of mCherry fluorescence, growth arrest as cessation of cell division and filamentation as a marked increase in cell length. Scale bars represent 1 µm. (*b*) Fraction of surviving cells as a function of time. MS2 induced lysis in 35.6% of 289 wt cells (orange) and in 40.9% of 428 Δ*mazEF* cells (blue). (*c*) Qβ induced lysis in 54.3% of 199 wt cells and in 55.3% of 329 Δ*mazEF* cells. Pale lines show individual replicates (three for MS2 and two for Qβ). Bold lines show averages across replicates. (*d*) Distributions of times to lysis for individual cells challenged with MS2 or (*e*) with Qβ. Horizontal lines show population averages (Mann–Whitney test, **p = 1.6 × 10^−5^).

To observe individual cell behaviour potentially hidden at the population level, we determined the fate of *E. coli* cells during RNA phage infection using a microfluidic device coupled with a fluorescence microscope [38]. *E. coli* cells constitutively expressing an *mCherry* fluorescent reporter were grown in narrow growth channels allowing a fast switch from phage-free to phage-containing medium (figure 2*a*).

In agreement with the mild effect observed in population-level measurements, native *mazEF* presence reduced single-cell lysis probability by 5.25% and 1.05% for MS2 and Qβ, respectively (figure 2*b*,*c*). A more profound effect was noticed when quantifying the time to cell lysis, as *mazEF* presence extended the average lysis times by 23% and 19% for MS2 and Qβ, respectively (figure 2*d*,*e*). Of the bacteria that survived phage exposure, a small fraction formed filaments and/or exhibited growth arrest during phage exposure, irrespective of *mazEF* presence (electronic supplementary material, figure S6 and movies S1 and S2). Finally, we observed that phage exposure increased the length of wt cells by 4% and 7% on average for MS2 and Qβ infection, respectively. In contrast, no significant increase was detected for Δ*mazEF* cells (electronic supplementary material, figure S7). Although phage infection can promote filament formation and destabilize the cell wall [39–42], further research is needed to clarify the relationship between antiphage systems and bacterial morphological changes [43].

### (c) RNA phage genomes exhibit bias against ACA sequences

To extend our analysis beyond the context of the MS2 and Qβ model systems, we investigated the abundance of the MazF recognition sequence ACA in sequenced RNA phage genomes infecting *E. coli*. For sequence-specific double-stranded DNA phage defence systems such as restriction–modification and CRISPR-Cas, avoidance of recognition sequences is common due to selection for reduced cleavage probability [44–47]. To test whether ACA trinucleotides are similarly avoided in RNA phage genomes, we first analysed reference genomes from the NCBI Viral Genomes Database. This dataset includes 12 RNA phage species with known bacterial hosts, i.e. phages infecting *Escherichia* sp., *Pseudomonas aeruginosa*, *Caulobacter crescentus* and *Acinetobacter baumannii*, as well as a broad host-range phage infecting *Escherichia* and *Pseudomonas* [48]. Notably, *mazF* homologues have been identified in *P. aeruginosa* and *A. baumannii*, even though their sequence specificity has not been determined [49,50].

**Figure 3.**
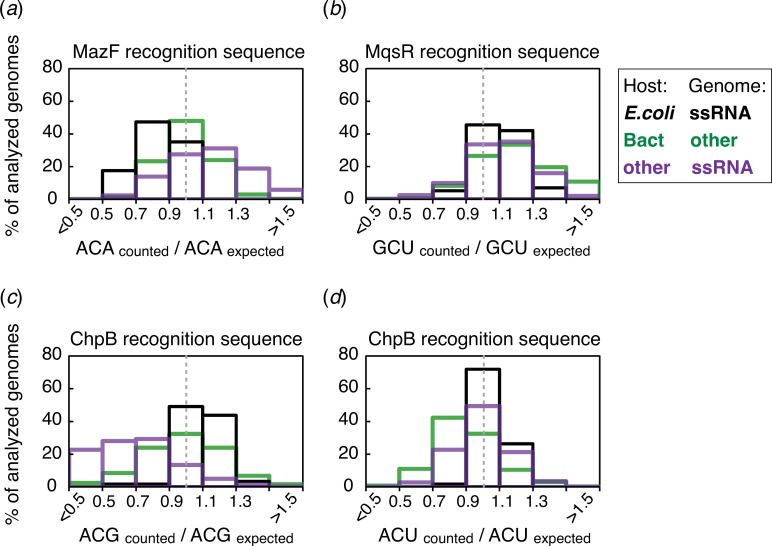
Relative frequencies of toxin recognition sequences in viral genomes. Relative frequencies were calculated as the actual recognition site count divided by the expected count. Black, green and purple bins show relative frequencies in (+)ssRNA phages infecting *E. coli* (*n* = 57), phages that do not have (+)ssRNA genomes (*n* = 2216) and (+)ssRNA viruses infecting non-bacterial hosts (*n* = 1835), respectively. Dashed grey lines depict the relative frequency of 1 (no bias). Relative frequencies of (*a*) ACA, (*b*) GCU, (*c*) ACG and (*d*) ACU trinucleotides in the genomes of RNA phages that infect *E. coli* were 0.83, 1.11, 1.09 and 1.03 on average, respectively.

The relative ACA frequency in the nine reference genomes of the RNA phages infecting *Escherichia* was in the range of 0.59 to 0.92, with an average of 0.76 (table 1). An extended analysis of 57 partial and complete RNA phage genomes infecting *Escherichia* likewise showed an underrepresentation of the ACA trinucleotide, with the mean relative frequency of 0.83 (figure 3*a*; electronic supplementary material, table S2). In contrast, phages that do not have ssRNA genomes and ssRNA viruses infecting non-bacterial hosts did not show a significant underrepresentation of the ACA trinucleotide. Additionally, random nucleotide-wise shuffling of the RNA phage genomes resulted in a significantly higher ACA frequency than in the actual phage genomes (table 1; electronic supplementary material, table S2 and supplementary datasets). Collectively, these results support the hypothesis that the ACA trinucleotide is less frequent than expected across RNA phage genomes.

**Table 1.** Bioinformatic analysis of RNA phage genomes from the NCBI reference sequence database.

				analysis of the genome				randomly shuffled genomes (10 000 runs) % of shuffled genomes with the number of ACA sites higher than in the original genome
phage name	phage species	pilus-encoding gene cluster^a^	accession number	genome length (nt)	number of ACA sites	number of expected ACA sites	relative ACA frequency	
MS2	*Emesvirus zinderi*	F plasmid-specific Group I	NC_001417.2	3569	47	51	0.92	69.75
BZ13 (GA)	*Emesvirus japonicum*	Group II	NC_001426.1	3466	32	50	0.64	99.75
Qβ (MX1)	*Qubevirus durum*	Group III	NC_001890.1	4215	53	59	0.90	76.03
FI 4184 b	*Qubevirus faecium*	Group IV	NC_028902.1	4184	44	56	0.78	95.52
FI *sensu lato* (SP)	*Qubevirus faecium*	Group IV	NC_004301.1	4276	47	62	0.76	97.43
C-1 INW-2012	*Cunavirus pretoriense*	IncC-specific	NC_019920.1	3523	35	52	0.68	99.50
Hgal1	*Hagavirus psychrophilum*	IncH-specific	NC_019922.1	3562	32	54	0.59	99.94
M	*Empivirus allolyticum*	IncM-specific	NC_019707.1	3405	42	47	0.89	76.65
broad host phage PRR1	*Perrunavirus olsenii*	IncP-specific	NC_008294.1	3573	38	54	0.70	98.70
*Pseudomonas* phage PP7	*Pepevirus rubrum*	plasmid- independent	NC_001628.1	3588	38	45	0.85	82.92
*Caulobacter* phage phiCb5	*Cebevirus halophobicum*	plasmid-independent	NC_019453.1	3762	32	52	0.62	99.92
*Acinetobacter* phage AP205	*Apeevirus quebecense*	plasmid-independent	NC_002700.2	4268	71	68	1.04	32.82

^a^ RNA phages infect bacterial cells by adsorbing to F-pili encoded on the conjugation F plasmid, to pili encoded on plasmids of different incompatibility (Inc) groups or to chromosome-encoded (plasmid-independent) pili.

Besides MazF, two other ribosome-independent sequence-specific type II toxins are known to be present in *E. coli*: MqsR that cleaves RNA at GCU sites, and ChpB that cleaves RNA at ACD sites (D is G, A or U, but not C) [6,51]. Even though ChpB is structurally and biochemically similar to MazF, it is a less processive enzyme than MazF. Unlike the ACA trinucleotide recognized by MazEF, the GCU, ACG and ACU trinucleotides were not underrepresented in the genomes of RNA phages infecting *E. coli* (figure 3*b*–*d*). This lack of recognition site avoidance could be either due to a lower selection pressure, for example, due to reduced activity of the corresponding toxins, or various evolutionary constraints that make particular sites difficult to mutate.

## 4. Discussion

Studies on how bacteria defend themselves against RNA phages are still relatively scarce and mostly rely on *in vitro* assays or non-physiological conditions [52–56]. Our findings show that the TA module *mazEF* provides moderate but measurable protection of *E. coli* against RNA phages *in vivo* under physiological conditions. MazEF system could protect *E. coli* in two ways: specifically, through MazF-mediated cleavage of phage RNA [17], and non-specifically, through MazF-mediated cellular RNA degradation followed by a reduction in overall translation and subsequent bacterial growth suppression [28], which can prevent phage amplification. Our competition and single-cell experiments indicate that direct interference, rather than abortive infection, plays the predominant role in the case of MazEF. However, this can vary among different TA systems.

In general, at least two possible mechanisms could lead to RNA being targeted by the active MazF toxin. First, phage-induced stress responses might disrupt the MazE antitoxin, thereby freeing MazF to cleave RNA. Alternatively, low-level stochastic fluctuations in TA dynamics [28] could transiently activate MazF independent of infection. Further research into the responses of TA systems during phage infection will be necessary to distinguish between these potential mechanisms.

While the effect of MazEF was consistently detected across multiple assays, its size was moderate at best. This is understandable, given that RNA phages, which initiate infection by adsorbing to bacterial F-pili, are less efficient viruses compared to DNA phages that adsorb to less complex structures [57,58]. Synthesis of F-pili is known to be highly heterogeneous across individual bacterial cells [59], and has been previously shown to be perturbed by the presence of RNA phages [60]. This reduces the fraction of cells that can be infected by the RNA phages and thus also the fraction in which the MazEF defence can manifest. In our experiments, MS2 and Qβ decreased the bacterial population size by approximately an order of magnitude, establishing an upper limit on the maximum achievable efficiency of any defence system in this context. In addition, RNA phages have been largely overlooked in research [33,61,62], and currently unknown antiphage mechanisms might still be at play in both wt and Δ*mazEF* strains.

Since all phages require RNA synthesis to complete their life cycle, the protection conferred by *mazEF* is not necessarily limited to ssRNA phages. Indeed, TA systems, such as RnlAB, LsoAB and MazEF, can also play a role in defence against DNA phages [63,64]. Specifically, the MazEF system of *E. coli* interacts with DNA phages P1 [65], λ [66] and T4 [67], although this might depend on the strain genotype [68]. Our study posits that TA systems may also serve as an initial line of defence against RNA phages. In support of this proposition, phages have evolved mechanisms to avoid TA-based antiphage strategies [63,67]. Our analysis builds on these studies in that it indicates that ACA sites in the RNA genomes of phages infecting *E. coli* are likely selected against, possibly because phages with fewer ACA sites are more likely to evade MazF action. Interestingly, while RNA-degrading activity can suppress gene expression in DNA phages, in the case of RNA phages, it could do both, suppress gene expression and eliminate the infecting RNA genome.

While the necessity to investigate RNA viruses in-depth has been recently recognized [69,70], our knowledge of the genomic diversity and host range of RNA phages remains vastly underestimated [33,61,62]. In many ways, research into RNA phage biology is still in its infancy, and identifying bacterial defence mechanisms against RNA phages will be essential for a more comprehensive understanding of prokaryotic biology.

## Data Availability

Electronic supplementary material is available online, figures S1–S7, tables S1–S2, movies S1–S2, supplementary methods and supplementary datasets (source data for experimental results and bioinformatic analysis). Time-lapse microscopy data are available from the Dryad Digital Repository [71]. Supplementary material is available online [72].

## References

[1] van Houte S, Buckling A, Westra ER. 2016 Evolutionary ecology of prokaryotic immune mechanisms. Microbiol. Mol. Biol. Rev. **80**, 745–763. (10.1128/mmbr.00011-16) 27412881 PMC4981670

[2] Doron S, Melamed S, Ofir G, Leavitt A, Lopatina A, Keren M, Amitai G, Sorek R. 2018 Systematic discovery of antiphage defense systems in the microbial pangenome. Science **359**, r4120. (10.1126/science.aar4120) PMC6387622 29371424

[3] Rostøl JT, Marraffini L. 2019 (Ph)ighting phages: how bacteria resist their parasites. Cell Host Microbe **25**, 184–194. (10.1016/j.chom.2019.01.009) 30763533 PMC6383810

[4] Bernheim A, Sorek R. 2020 The pan-immune system of bacteria: antiviral defence as a community resource. Nat. Rev. Microbiol. **18**, 113–119. (10.1038/s41579-019-0278-2) 31695182

[5] Vassallo CN, Doering CR, Littlehale ML, Teodoro GIC, Laub MT. 2022 A functional selection reveals previously undetected anti-phage defence systems in the E. coli pangenome. Nat. Microbiol. **7**, 1568–1579. (10.1038/s41564-022-01219-4) 36123438 PMC9519451

[6] Gerdes K (ed). 2012 Prokaryotic toxin–antitoxins. Berlin, Germany: Springer. (10.1007/978-3-642-33253-1)

[7] Mets T *et al*. 2017 Toxins MazF and MqsR cleave Escherichia coli rRNA precursors at multiple sites. RNA Biol. **14**, 124–135. (10.1080/15476286.2016.1259784) 27858580 PMC5270532

[8] LeRoux M, Culviner PH, Liu YJ, Littlehale ML, Laub MT. 2020 Stress can induce transcription of toxin–antitoxin systems without activating toxin. Mol. Cell **79**, 280–292.(10.1016/j.molcel.2020.05.028) 32533919 PMC7368831

[9] Van Melderen L. 2010 Toxin–antitoxin systems: why so many, what for? Curr. Opin. Microbiol. **13**, 781–785. (10.1016/j.mib.2010.10.006) 21041110

[10] Nikolic N, Didara Z, Moll I. 2017 MazF activation promotes translational heterogeneity of the grcA mRNA in Escherichia coli populations. PeerJ **5**, e3830. (10.7717/peerj.3830) 28948108 PMC5610899

[11] Nikolic N, Sauert M, Albanese TG, Moll I. 2022 Quantifying heterologous gene expression during ectopic MazF production in Escherichia coli. BMC Res. Notes **15**. (10.1186/s13104-022-06061-9) PMC9102682 35562780

[12] Song S, Wood TK. 2020 A primary physiological role of toxin/antitoxin systems is phage inhibition. Front. Microbiol. **11**, 1895. (10.3389/fmicb.2020.01895) 32903830 PMC7438911

[13] Kelly A, Arrowsmith TJ, Went SC, Blower TR. 2023 Toxin–antitoxin systems as mediators of phage defence and the implications for abortive infection. Curr. Opin. Microbiol. **73**, 102293. (10.1016/j.mib.2023.102293) 36958122

[14] Norton JP, Mulvey MA. 2012 Toxin–antitoxin systems are important for niche-specific colonization and stress resistance of uropathogenic Escherichia coli. PLoS Pathog. **8**, e1002954. (10.1371/journal.ppat.1002954) 23055930 PMC3464220

[15] Fiedoruk K, Daniluk T, Swiecicka I, Sciepuk M, Leszczynska K. 2015 Type II toxin–antitoxin systems are unevenly distributed among Escherichia coli phylogroups. Microbiology **161**, 158–167. (10.1099/mic.0.082883-0) 25378561

[16] Zhang Y, Zhang J, Hoeflich KP, Ikura M, Qing G, Inouye M. 2003 MazF cleaves cellular mRNAs specifically at ACA to block protein synthesis in Escherichia coli. Mol. Cell **12**, 913–923. (10.1016/s1097-2765(03)00402-7) 14580342

[17] Zorzini V, Mernik A, Lah J, Sterckx YGJ, De Jonge N, Garcia-Pino A, De Greve H, Versées W, Loris R. 2016 Substrate recognition and activity regulation of the Escherichia coli mRNA endonuclease MazF. J. Biol. Chem. **291**, 10950–10960. (10.1074/jbc.m116.715912) 27026704 PMC4900246

[18] Culviner PH, Laub MT. 2018 Global analysis of the E. coli toxin MazF reveals widespread cleavage of mRNA and the inhibition of rRNA maturation and ribosome biogenesis. Mol. Cell **70**, 868–880.(10.1016/j.molcel.2018.04.026) 29861158 PMC8317213

[19] van Duin JV, Tsareva N, 2006. Single-stranded RNA phages. In The bacteriophages (ed. RL Calendar), pp. 175–196, 2nd edn. Oxford, UK: Oxford University Press. (10.1093/oso/9780195148503.003.0015)

[20] Olsthoorn RC, Licis N, van Duin J. 1994 Leeway and constraints in the forced evolution of a regulatory RNA helix. EMBO J. **13**, 2660–2668. (10.1002/j.1460-2075.1994.tb06556.x) 8013465 PMC395140

[21] Klovins J, Berzins V, van Duin J. 1998 A long-range interaction in Qβ RNA that bridges the thousand nucleotides between the M-site and the 3′ end is required for replication. RNA **4**, 948–957. (10.1017/s1355838298980177) 9701286 PMC1369672

[22] Nariya H, Inouye M. 2008 MazF, an mRNA interferase, mediates programmed cell death during multicellular Myxococcus development. Cell **132**, 1. (10.1016/j.cell.2007.11.044) 18191220

[23] Zhu L, Phadtare S, Nariya H, Ouyang M, Husson RN, Inouye M. 2008 The mRNA interferases, MazF‐mt3 and MazF‐mt7 from Mycobacterium tuberculosis target unique pentad sequences in single‐stranded RNA. Mol. Microbiol. **69**, 559–569. (10.1111/j.1365-2958.2008.06284.x) 18485066 PMC6291307

[24] Zhu L, Inoue K, Yoshizumi S, Kobayashi H, Zhang Y, Ouyang M, Kato F, Sugai M, Inouye M. 2009 Staphylococcus aureus MazF specifically cleaves a pentad sequence, UACAU, which is unusually abundant in the mRNA for pathogenic adhesive factor SraP. J. Bacteriol. **191**, 3248–3255. (10.1128/jb.01815-08) 19251861 PMC2687152

[25] Park JH, Yamaguchi Y, Inouye M. 2011 Bacillus subtilis MazF‐bs (EndoA) is a UACAU‐specific mRNA interferase. FEBS Lett. **585**, 2526–2532. (10.1016/j.febslet.2011.07.008) 21763692 PMC3167231

[26] Leon D, D’Alton S, Quandt EM, Barrick JE. 2018 Innovation in an E. coli evolution experiment is contingent on maintaining adaptive potential until competition subsides. PLoS Genet. **14**, e1007348. (10.1371/journal.pgen.1007348) 29649242 PMC5918244

[27] Bergmiller T, Andersson AMC, Tomasek K, Balleza E, Kiviet DJ, Hauschild R, Tkačik G, Guet CC. 2017 Biased partitioning of the multidrug efflux pump AcrAB-TolC underlies long-lived phenotypic heterogeneity. Science **356**, 311–315. (10.1126/science.aaf4762) 28428424

[28] Nikolic N, Bergmiller T, Vandervelde A, Albanese TG, Gelens L, Moll I. 2018 Autoregulation of mazEF expression underlies growth heterogeneity in bacterial populations. Nucleic Acids Res. **46**, 2918–2931. (10.1093/nar/gky079) 29432616 PMC5888573

[29] Chait R, Ruess J, Bergmiller T, Tkačik G, Guet CC. 2017 Shaping bacterial population behavior through computer-interfaced control of individual cells. Nat. Commun. **8**. (10.1038/s41467-017-01683-1) PMC5688142 29142298

[30] May T, Tsuruta K, Okabe S. 2011 Exposure of conjugative plasmid carrying Escherichia coli biofilms to male-specific bacteriophages. ISME J. **5**, 771–775. (10.1038/ismej.2010.158) 20962879 PMC3105745

[31] Brister JR, Ako-adjei D, Bao Y, Blinkova O. 2015 NCBI viral genomes resource. Nucleic Acids Res. **43**, D571–D577. (10.1093/nar/gku1207) 25428358 PMC4383986

[32] Friedman SD, Genthner FJ, Gentry J, Sobsey MD, Vinjé J. 2009 Gene mapping and phylogenetic analysis of the complete genome from 30 single-stranded RNA male-specific coliphages (family Leviviridae). J. Virol. **83**, 11233–11243. (10.1128/jvi.01308-09) 19710143 PMC2772794

[33] Krishnamurthy SR, Janowski AB, Zhao G, Barouch D, Wang D. 2016 Hyperexpansion of RNA bacteriophage diversity. PLoS Biol. **14**, e1002409. (10.1371/journal.pbio.1002409) 27010970 PMC4807089

[34] Rappaport I. 1965 Some studies of the infectious process with MS2 bacteriophage. Biochim. Biophys. Acta Nucleic Acids Protein Synth. **103**, 486–494. (10.1016/0005-2787(65)90141-3) 5853494

[35] Jenkins ST, Beard JP, Howe TGB. 1974 Male-specific bacteriophage MS2 propagation in fluorophenylalanine-resistant Escherichia coli K12. J. Virol. **14**, 1. (10.1128/jvi.14.1.50-55.1974) 4599509 PMC355477

[36] Woody MA, Cliver DO. 1995 Effects of temperature and host cell growth phase on replication of F-specific RNA coliphage Q beta. Appl. Environ. Microbiol. **61**, 1520–1526. (10.1128/aem.61.4.1520-1526.1995) 7747969 PMC167408

[37] Tsukada K, Okazaki M, Kita H, Inokuchi Y, Urabe I, Yomo T. 2009 Quantitative analysis of the bacteriophage Qβ infection cycle. Biochim. Biophys. Acta Gen. Subj. **1790**, 65–70. (10.1016/j.bbagen.2008.08.007) 18790012

[38] Wang P, Robert L, Pelletier J, Dang WL, Taddei F, Wright A, Jun S. 2010 Robust growth of Escherichia coli. Curr. Biol. **20**, 1099–1103. (10.1016/j.cub.2010.04.045) 20537537 PMC2902570

[39] Attrill EL, Claydon R, Łapińska U, Recker M, Meaden S, Brown AT, Westra ER, Harding SV, Pagliara S. 2021 Individual bacteria in structured environments rely on phenotypic resistance to phage. PLoS Biol. **19**, e3001406. (10.1371/journal.pbio.3001406) 34637438 PMC8509860

[40] Attrill EL, Łapińska U, Westra ER, Harding SV, Pagliara S. 2023 Slow growing bacteria survive bacteriophage in isolation. ISME Commun. **3**, 95. (10.1038/s43705-023-00299-5) 37684358 PMC10491631

[41] Soonthonsrima T, Htoo HH, Thiennimitr P, Srisuknimit V, Nonejuie P, Chaikeeratisak V. 2023 Phage-induced bacterial morphological changes reveal a phage-derived antimicrobial affecting cell wall integrity. Antimicrob. Agents Chemother. **67**, e0076423. (10.1128/aac.00764-23) 37843261 PMC10648931

[42] Dhanoa GK, Kushnir I, Qimron U, Roper DI, Sagona AP. 2022 Investigating the effect of bacteriophages on bacterial FtsZ localisation. Front. Cell. Infect. Microbiol. **12**, 863712. (10.3389/fcimb.2022.863712) 35967845 PMC9372555

[43] Zhang Z *et al*. 2025 Kiwa is a bacterial membrane-embedded defence supercomplex activated by phage-induced membrane changes. bioRxiv. (10.1101/2023.02.26.530102) PMC12348683 40730155

[44] Sharp PM. 1986 Molecular evolution of bacteriophages: evidence of selection against the recognition sites of host restriction enzymes. Mol. Biol. Evol. **3**, 75–83. (10.1093/oxfordjournals.molbev.a040377) 2832688

[45] Kupczok A, Bollback JP. 2014 Motif depletion in bacteriophages infecting hosts with CRISPR systems. BMC Genom. **15**, 663. (10.1186/1471-2164-15-663) PMC4246573 25103210

[46] Pleška M, Qian L, Okura R, Bergmiller T, Wakamoto Y, Kussell E, Guet CC. 2016 Bacterial autoimmunity due to a restriction–modification system. Curr. Biol. **26**, 404–409. (10.1016/j.cub.2015.12.041) 26804559

[47] Pleška M, Guet CC. 2017 Effects of mutations in phage restriction sites during escape from restriction–modification. Biol. Lett. **13**, 20170646. (10.1098/rsbl.2017.0646) 29237814 PMC5746541

[48] Olsen RH, Shipley P. 1973 Host range and properties of the Pseudomonas aeruginosa R factor R1822. J. Bacteriol. **113**, 772–780. (10.1128/jb.113.2.772-780.1973) 4632321 PMC285292

[49] Jurenaite M, Markuckas A, Suziedeliene E. 2013 Identification and characterization of type II toxin–antitoxin systems in the opportunistic pathogen Acinetobacter baumannii. J. Bacteriol. **195**, 3165–3172. (10.1128/JB.00237-13) 23667234 PMC3697630

[50] Valadbeigi H, Sadeghifard N, Salehi MB. 2017 Assessment of biofilm formation in Pseudomonas aeruginosa by antisense mazE-PNA. Microb. Pathog. **104**, 28–31. (10.1016/j.micpath.2017.01.009) 28062294

[51] Masuda H, Inouye M. 2017 Toxins of prokaryotic toxin–antitoxin systems with sequence-specific endoribonuclease activity. Toxins **9**, 140. (10.3390/toxins9040140) 28420090 PMC5408214

[52] Klovins J, van Duin J, Olsthoorn RCL. 1997 Rescue of the RNA phage genome from RNase III cleavage. Nucleic Acids Res. **25**, 4201–4208. (10.1093/nar/25.21.4201) 9336447 PMC147046

[53] Abudayyeh OO *et al*. 2016 C2c2 is a single-component programmable RNA-guided RNA-targeting CRISPR effector. Science **353**, aaf5573. (10.1126/science.aaf5573) 27256883 PMC5127784

[54] Smargon AA *et al*. 2017 Cas13b is a type VI-B CRISPR-associated RNA-guided RNase differentially regulated by accessory proteins Csx27 and Csx28. Mol. Cell **65**, 618–630.(10.1016/j.molcel.2016.12.023) 28065598 PMC5432119

[55] Strutt SC, Torrez RM, Kaya E, Negrete OA, Doudna JA. 2018 RNA-dependent RNA targeting by CRISPR-Cas9. eLife **7**, e32724. (10.7554/elife.32724) 29303478 PMC5796797

[56] Yan WX *et al*. 2019 Functionally diverse type V CRISPR-Cas systems. Science **363**, 88–91. (10.1126/science.aav7271) 30523077 PMC11258546

[57] Nikolic N, Anagnostidis V, Tiwari A, Chait R, Gielen F. 2023 Droplet-based methodology for investigating bacterial population dynamics in response to phage exposure. Front. Microbiol. **14**, 1260196. (10.3389/fmicb.2023.1260196) 38075890 PMC10703435

[58] Tiwari A, Nikolic N, Anagnostidis V, Gielen F. 2023 Label-free analysis of bacterial growth and lysis at the single-cell level using droplet microfluidics and object detection-oriented deep learning. Front. Lab Chip Technol. **2**, 1258155. (10.3389/frlct.2023.1258155)

[59] Biebricher CK, Düker EM. 1984 F and type 1 piliation of Escherichia coli. Microbiology **130**, 951–957. (10.1099/00221287-130-4-951) 6145751

[60] Harb L, Chamakura K, Khara P, Christie PJ, Young R, Zeng L. 2020 ssRNA phage penetration triggers detachment of the F-pilus. Proc. Natl. Acad. Sci. USA **117**, 25751–25758. (10.1073/pnas.2011901117) 32989140 PMC7568308

[61] Callanan J, Stockdale S, Shkoporov A, Draper L, Ross R, Hill C. 2018 RNA phage biology in a metagenomic era. Viruses **10**, 386. (10.3390/v10070386) 30037084 PMC6071253

[62] Neri U *et al*. 2022 Expansion of the global RNA virome reveals diverse clades of bacteriophages. Cell **185**, 4023–4037. (10.1016/j.cell.2022.08.023) 36174579

[63] Otsuka Y. 2016 Prokaryotic toxin–antitoxin systems: novel regulations of the toxins. Curr. Genet. **62**, 379–382. (10.1007/s00294-015-0557-z) 26780368

[64] Jurėnas D, Fraikin N, Goormaghtigh F, Van Melderen L. 2022 Biology and evolution of bacterial toxin–antitoxin systems. Nat. Rev. Microbiol. **20**, 335–350. (10.1038/s41579-021-00661-1) 34975154

[65] Hazan R, Engelberg-Kulka H. 2004 Escherichia coli mazEF-mediated cell death as a defense mechanism that inhibits the spread of phage P1. Mol. Genet. Genom. **272**, 227–234. (10.1007/s00438-004-1048-y) 15316771

[66] Engelberg‐Kulka H, Kumar S. 2015 Yet another way that phage λ manipulates its Escherichia coli host: λ rexB is involved in the lysogenic–lytic switch. Mol. Microbiol. **96**, 689–693. (10.1111/mmi.12969) 25684601

[67] Alawneh AM, Qi D, Yonesaki T, Otsuka Y. 2016 An ADP‐ribosyltransferase Alt of bacteriophage T4 negatively regulates the Escherichia coli MazF toxin of a toxin–antitoxin module. Mol. Microbiol. **99**, 188–198. (10.1111/mmi.13225) 26395283

[68] Guegler CK, Laub MT. 2021 Shutoff of host transcription triggers a toxin–antitoxin system to cleave phage RNA and abort infection. Mol. Cell **81**, 2361–2373.(10.1016/j.molcel.2021.03.027) 33838104 PMC8284924

[69] Dominguez-Huerta G *et al*. 2022 Diversity and ecological footprint of global ocean RNA viruses. Science **376**, 1202–1208. (10.1126/science.abn6358) 35679415

[70] Zayed AA *et al*. 2022 Cryptic and abundant marine viruses at the evolutionary origins of Earth’s RNA virome. Science **376**, 156–162. (10.1126/science.abm5847) 35389782 PMC10990476

[71] Nikolic N, Pleška M, Bergmiller T, Guet C. 2025 A bacterial toxin-antitoxin system as a native defence element against RNA phages. Dryad Digital Repository. (10.5061/dryad.zgmsbccq5)40494395

[72] Nikolic N, Pleška M, Bergmiller T, Guet CC. 2025 Supplementary material from: A bacterial toxin-antitoxin system as a native defence element against RNA phages. Figshare. (10.6084/m9.figshare.c.7859199)40494395

